# Weakened Resilience in Parenting Self-Efficacy in Pregnant Women Who Were Abused in Childhood: An Experimental Test

**DOI:** 10.1371/journal.pone.0141801

**Published:** 2016-02-05

**Authors:** Florentina C. Kunseler, Mirjam Oosterman, Marleen H. M. de Moor, Marije L. Verhage, Carlo Schuengel

**Affiliations:** 1 Section of Clinical Child and Family Studies, Vrije Universiteit Amsterdam, The Netherlands; 2 EMGO+ Institute for Health and Care Research, Vrije Universiteit Amsterdam, The Netherlands; University of California, San Francisco, UNITED STATES

## Abstract

This study tested experimentally whether the combination of a history of childhood abuse and confrontation with difficult infant temperament is associated with negative changes in parenting self-efficacy. First-time pregnant women (*N* = 243) participated in the Adult Attachment Interview, which was used to assess the occurrence of abuse by parents in childhood and unresolved representations, and completed a task asking them to respond to infant cries. Sixty of the 243 participants (25%) experienced childhood abuse, mostly physical or sexual. The task simulated infant temperamental difficulty by manipulating soothing success in order to reflect an easy-to-soothe (80% soothing success) and a difficult-to-soothe infant (20% soothing success). Both after baseline and after each of the two stimulus series women assessed their parenting self-efficacy. Women who reported childhood abuse did not differ from women who reported no childhood abuse in parenting self-efficacy at baseline or in response to the easy-to-soothe infant (relative to baseline), but decreased more in parenting self-efficacy following the difficult-to-soothe infant. Effects did not vary according to resolution of trauma. These findings suggest that in response to infant temperamental difficulty, women who experienced childhood abuse may more easily lose confidence in their parenting abilities, which underlines the importance of preparing at-risk women for the possible challenges that come along with parenthood.

## Introduction

Parenting self-efficacy (PSE), defined as “the expectation caregivers hold about their ability to parent successfully” ([1], p. 342) is an important indicator of women’s adaptation to parenthood [2, 3]. Before women have their child and actually build up experience, they already hold expectations about their parenting competence, which at that point may be based, among other sources, on their evaluations of own childhood experiences [4, 5]. Preliminary support for a negative relation between childhood rejection or abuse and PSE comes from a limited number of studies mostly indicative of small effects and mixed with regard to finding direct or indirect relations [6–9]. More generally, research on the intergenerational transmission of risk suggests large individual differences between people as to whether their experience of childhood abuse affects outcomes, such as abusing one’s own children [10]. The individual differences may partly result from the combination of personal risk factors and stressors within the actual child rearing setting itself, such as difficult temperament of the newborn. However, the interaction between maternal and child characteristics in the adaptation to parenthood is difficult to study. Child characteristics are mostly measured by parents’ perceptions, which may be determined by parental self-perceptions as well as actual parenting behavior [11, 12]. To mitigate this problem, the current study used an experimental design with standardized child stimuli during pregnancy to test whether the combination of a history of childhood abuse and confrontation with difficult infant temperament would lead to more negative changes in PSE.

On the basis of interactions with parents or caregivers children develop working models about themselves and their relationships, which play an important role in the formation of social beliefs, expectations, and behaviors, including parenting beliefs, expectations, and behaviors [4, 5, 13]. Leerkes and Crockenberg [8] found that primiparous women who rated their parents as accepting and warm in childhood had higher self-esteem and higher prenatal expectations of their parenting competence. In contrast, maternal reports of childhood abuse were found to be negatively linked to PSE [6, 7, 14]. These studies on the consequences of childhood abuse measured PSE at one time point in the postpartum period only, which makes it difficult to distinguish whether women exposed to childhood abuse have stable low scores of PSE over time or developed a lower sense of postpartum PSE in response to parenting challenges or failures.

Infant distress is one of the challenges that parents are exposed to in the transition to parenthood. Features of infant difficult temperament, such as frequent crying or low soothability, may be stressful to new parents. Several studies found a negative association between PSE and infant difficult temperament [8, 15–17], consistent with the theoretical assumptions of Bandura [2, 18] that failure experiences may lower people’s sense of efficacy, whereas success experiences enhance self-efficacy. Results of a study by Verhage, Oosterman, and Schuengel [19] revealed that women already adapted their sense of parenting competence to successes and failures in soothing a crying infant during pregnancy. Women who were abused as a child may be even more inclined to negatively adjust their PSE in response to failures in soothing, because infant distress and unsuccessful regulation of distress may be interpreted within a general framework of negative expectations and low self-esteem [8, 9]. That way, a history of childhood adversity and infant difficult temperament may interact to create lower appraisals of parenting efficacy, which was found by Crockenberg and Leerkes for depressive symptoms [20]. In addition, women who reported abuse experiences may have built up less trust in their abilities to handle demanding activities, because they missed positive parental modeling in childhood [2, 8]. Other research suggest that infant crying elicits stronger emotional and physiological responses in parents who are at risk to abuse their child [21, 22], as evidenced by more hostility, more negative affect and higher heart rates. Such a hyperreactive response pattern may be associated with decreased parenting self-efficacy as well [18].

Although the main focus of this study is on the association between maternal abuse experiences and PSE, attachment research has demonstrated associations between *unresolved mental representations of abuse experiences* and parenting [23]. Unresolved trauma can be classified with the Adult Attachment Interview (AAI), which aims to assess adults’ mental representation or state of mind with respect to attachment. The AAI covers experiences with primary caregivers in childhood and effects of early experiences on current functioning. Attention is also paid to experiences with loss, abuse or other trauma [24, 25]. A lack of resolution of trauma, which is conceptualized as a mental disorganization, may become evident in lapses in monitoring of reasoning or discourse when recounting these potential traumatic experiences during the interview ([24, 25] see Methods for more information about the AAI).

To our knowledge, no previous studies focused on differential responses to infant crying in adults with unresolved versus resolved abuse experiences. However, both disorganization with respect to trauma and PTSD were found to be associated with more limbic system activity and less effective regulation by the prefrontal cortex in response to other attachment related stressors, such as watching a parent-child separation or telling attachment-related stories [26–28]. This was suggested to be indicative of people’s emotion dysregulation and executive control processes being overwhelmed by distress associated with traumatic reminders [28]. On a physiological level, one study found no evidence for dysregulation in the unresolved group while they were responding to the AAI questions about loss or abuse [29]. However, in another study people with unresolved traumatic experiences showed heightened physiological arousal in response to watching parent-child separations and reunions [30, 31]. Although findings are scarce and not entirely consistent, neural and physiological evidence does suggest that women with unresolved abuse experiences may experience stronger reactivity to challenging parenting situations.

This study compared the adaptation of PSE of women with and without reported childhood abuse experiences to difficult infant behavior, specifically low soothability. An experimentally manipulated task previously described by Verhage and colleagues [19] was used, in which first-time pregnant women were asked to comfort an easy-to-soothe and a difficult-to-soothe infant, reflecting a success and a failure condition, respectively. A difficult soothing experience was assessed relative to an easy soothing experience to compare how women with or without abuse experiences would adjust their PSE in response to similar—low or high challenging- parenting situations. In addition, women’s adjustment in PSE to an easy soothing experience was examined (relative to PSE at baseline). Besides average levels of women’s adjustment of PSE, individual differences in this adjustment were examined. It was hypothesized that childhood abuse experiences could partly explain the individual differences in women’s adjustment of PSE in response to failures relative to successes in soothing a crying infant, in such a way that women who reported childhood abuse with (one of) their parent(s), would show stronger decreases in PSE compared to women who reported no childhood abuse experiences [2, 9]. Women who reported abuse were not expected to differently adjust their PSE to the easy-to-soothe infant in comparison to women who did not report abuse. Finally, it was explored to what extent unresolved representations of childhood abuse were associated with PSE.

## Method

### Participants

Women who were pregnant with their first child were recruited by midwifes, through a website, and by approaching visitors to a pregnancy fair for participation in a longitudinal cohort study on pregnancy and parenthood (Generations^2^) involving questionnaires from the first trimester of pregnancy to one year postpartum. From this longitudinal study, women were invited to participate in additional measurements (home visits) during pregnancy and the postpartum period if they had granted permission to be contacted for other measurements linked to the longitudinal study. Two subgroups were selected for the current report: an “at-risk” and a “normative” subgroup. For the at-risk subgroup, women were selected if they reported experiences with youth care or with a psychiatrist or psychologist before the age of 18 and for the normative subgroup, all women who lived in the vicinity of the research facility were approached. For the at-risk subgroup, first-time pregnant women were additionally approached from youth care facilities or institutions. Women were excluded for both subgroups if they had a prenatal diagnosis for a congenital abnormality of the fetus. Written informed consent was obtained from all participants. For minors participating in this study (under the age of 18 years) written informed consent was also obtained from parents and/or other legal guardians. The study has been approved by the Medical Ethical committee of the VU Medical Centre (METc), registration number NL24319.029.08.

Corresponding to the aim of the current study, the normative and the at-risk subgroup were combined in order to obtain a larger group of women who reported childhood abuse experiences. A total of 243 women participated in this study, of whom 142 originated from the normative subgroup and 101 from the at-risk subgroup. Fifty-five women of the at-risk study reported youth care or contact with a psychiatrist or psychologist before the age of 18, and 46 women were involved with (youth) care facilities or residing in youth care institutions at the moment of inclusion (response rate for both approximately 50%). For the combined group, women’s mean age was 28.18 (range 15–41, *SD* = 5.80). With respect to educational attainment, 142 women (58%) had a bachelor or master’s degree, 91 women (38%) finished high school or vocational training and 10 women (4%) finished primary education. Two hundred and ten women (86%) had a partner, of whom 195 women were married or cohabiting. Thirty-three women (14%) were single. Most of the women (73%) of the current sample were Dutch as based on their parent’s country of birth, 27 women (11%) had a non-Dutch Western background and 39 women (16%) a non-Western background.

### Procedure

Assessments took place at home. Women were on average 24.93 weeks pregnant (*SD* = 4.64; based on their due date) at the time of the home visit. A trained interviewer conducted the Adult Attachment Interview [32] and the Cry Response Task [19]. The computerized Cry Response Task, programmed in E-prime, was developed to assess women’s resilience of PSE in response to audio-taped infant crying sounds. Participants completed the 25-minute task on a laptop, which was placed in front of them on a table. As participants progressed through the task, instructions were provided on the screen.

The Cry Response Task [19] began with a 6-minute baseline with easy listening guitar-music and pictures of landscapes appearing on the screen. After the baseline, women were asked to assess their PSE. Then, women were instructed to listen to different infant cry sounds and choose one of four caregiving options to soothe the infant in response to each cry sound (e.g., pick the infant up, change diapers) or do nothing. The task consisted of two stimulus series each comprising ten different cry sounds. After each cry sound, “performance” feedback was provided to the women by means of a green or red smiley face, indicative of successful or unsuccessful soothing, respectively. In addition, the length of duration of the cries varied so that successful soothing was consistent with a cry sound duration of 15–20 seconds, and unsuccessful soothing with a cry sound duration of 30 seconds. The task was experimentally manipulated so that all women received 80% positive feedback with respect to the first ten cry sounds (baby 1 –“the easy-to-soothe infant”) and 20% positive feedback with respect to the second ten cry sounds (baby 2 –“the difficult-to-soothe infant”). Women were asked to fill out their PSE and cry perception separately after listening to the cry sounds of baby 1 and after the cry sounds of baby 2 (cry perception ratings were not used in the current study). After finishing the Cry Response Task, women were thoroughly debriefed about the manipulation.

### Instruments

#### Parenting self-efficacy

During the Cry Response Task PSE was measured three times with a Pictographic Visual Analogue Scale (VAS), following a design by Kalichman and colleagues [33]. A Visual Analogue Scale is suitable to examine fine grained changes in PSE also in lower literacy populations [33]. It was thought to be less distracting and disruptive from the task than other self-report measures that mostly consist of multiple questions, because women could report their PSE directly after listening to the cry sounds. Women were asked to answer the following question on a colored bar: “How well do you expect to respond to infant crying in daily situations?”. The bar changed from red at the left-hand side to green at the right hand side, with a thumbs down picture underneath the red and a thumbs-up picture underneath the green side of the bar, indicating lower to higher expectations respectively. Answers were registered by E-prime on a scale from 0 to 100. In a previous study, a Visual Analogue Scale was found to be a valid measure for self-efficacy, especially for task-specific self-efficacy [34]. In this study, PSE assessed with the VAS after the baseline was significantly associated with PSE as measured with the Self-Efficacy in the Nurturing Role questionnaire (SENR) [35], which was filled out by women during pregnancy (*r* (240) = .41, *p* < .001).

#### Abuse experiences with parents in childhood

To examine the occurrence of abuse experiences with parents in childhood and participant’s unresolved status with respect to these abuse experiences, the Dutch version of the Adult Attachment Interview [32] was used. This semi-structured interview contains 20 questions regarding people’s qualifications of their early relationship with their caregivers, their early and current experiences with them, and how these experiences relate to their adult personality. In addition, the interview includes questions on loss, abuse, and other traumatic experiences. The questions on abuse experiences included several behaviorally focused sub-questions to obtain more clarity on the occurrence or incidents of maltreatment if necessary, as was suggested by Bailey, Moran, and Pederson [36] and by Madigan, Vaillancourt, McKibbon, and Benoit [37]. All interviews were transcribed verbatim and rated with the Main and Goldwyn coding system [24] by certified coders. Although the main goal of the coding process is to operationalize people’s state of mind regarding attachment, for the current study we used only women’s reports of abuse by their parents or caregivers. The system primarily refers to physical maltreatment and sexual abuse, but also includes bizarre punishments of the child, parents’ attempts of suicide, or other frightening behaviors exhibited by parents in presence of the child [24], which were all considered as abuse in this study. Physical maltreatment is defined as any hitting by the caregiver leaving marks and repeated hitting that is hard and inappropriate or experienced by the child as particularly physically frightening. Any sexual experiences with parents or caregivers are defined as sexual abuse [24]. For part of the sample (*n* = 98), self-reported physical abuse scores measured with the Adverse Childhood Experiences (ACE) questionnaire ([38], see also http://acestudy.org/ace-score) were also available. The AAI and the ACE converged for 89% of the cases on the occurrence (yes/no) of physical abuse, of whom 26 reported physical abuse by (one of their) parents on both measures.

### Data analyses

Analyses for the current study were performed in SPSS version 20 and Mplus version 5.21 [39]. Preliminary analyses were performed in SPSS to examine the associations of abuse with demographic variables and the associations of abuse with PSE level at baseline, after exposure to the easy-to-soothe infant (baby1), and after exposure to the difficult to soothe infant (baby2).

Success and failure experiences are different processes that may underlie changes in PSE from baseline to the easy-to-soothe infant and from the easy-to-soothe infant to the difficult-to-soothe infant. Therefore, changes in PSE were examined in two separate latent growth curve models specified in Mplus from baseline to baby1 (model 1), and from baby1 to baby2 (model 2). With this technique, both mean changes in PSE and individual differences in changes of PSE were tested. Models were estimated using full information maximum likelihood.

**Fig 1:**
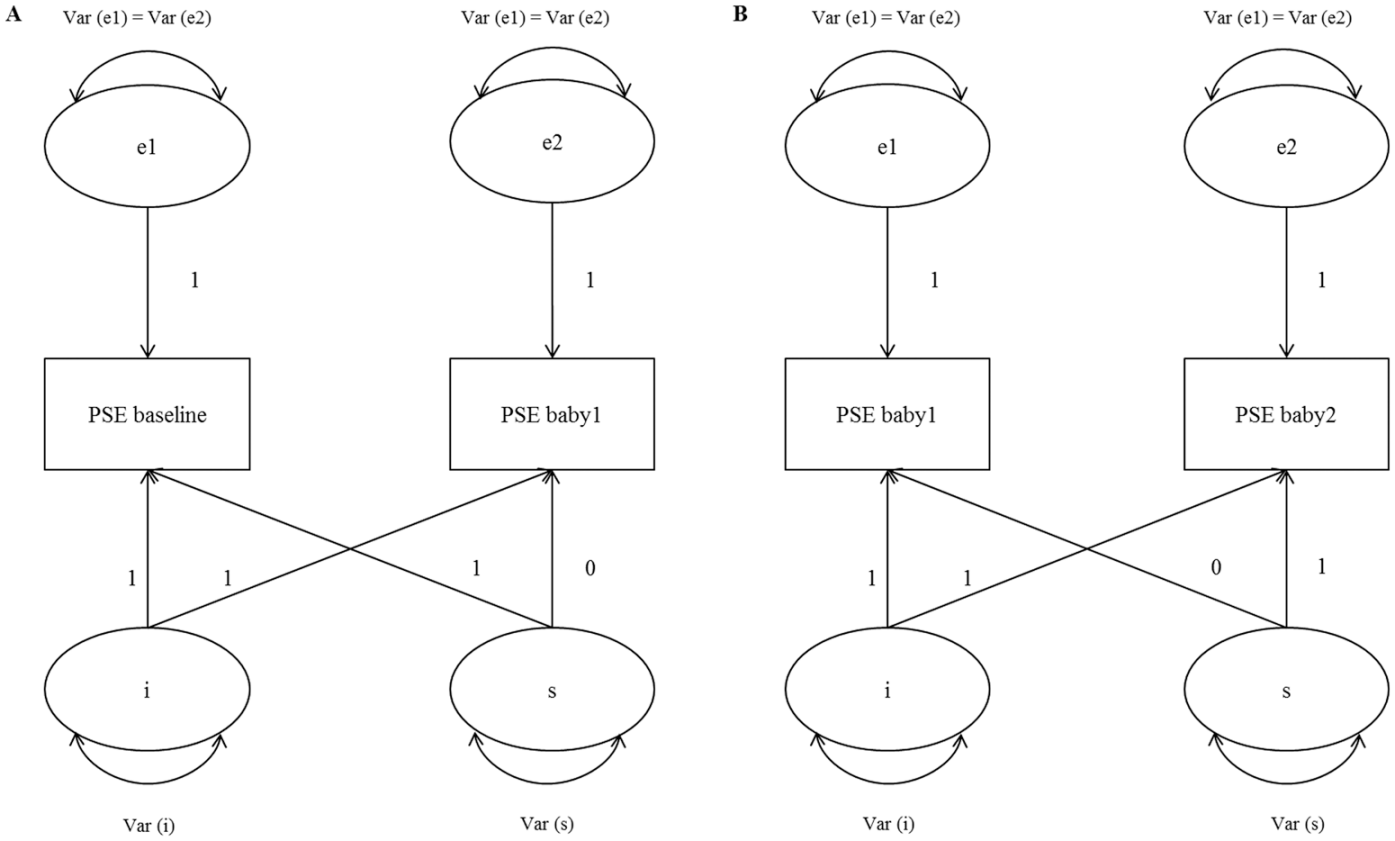
Fitted latent growth curve models for PSE during the Cry Response Task. (A) Model 1: Changes in PSE from baseline to baby 1. (B) Model 2: Changes in PSE from baby1 to baby2. PSE = Parenting Self-Efficacy; Var = variance; i = intercept; s = slope; e1 and e2 = residuals.

First, linear growth curve models were fitted for model 1 and model 2 separately (see Fig 1). Model 1 was specified for the two observed variables PSE baseline and PSE baby1 (Fig 1A) and model 2 was specified for the two observed variables PSE baby1 and PSE baby2 (Fig 1B). The first latent variable in both models indicated the intercept and the second latent variable indicated the slope. As can be observed in Fig 1A, the time scores for the slope growth factor of model 1 were coded in reversed order, 1 for baseline PSE and 0 for PSE baby1, to deal with a decrease in variance with respect to PSE scores from baseline to baby1. Please note that for model 1 the direction of associations therefore need to be reversed for interpretation, given the reversed time scores for the slope growth factor. Time scores for the slope growth factor of model 2 were coded as 0 for PSE baby1 and 1 for PSE baby2 respectively (Fig 1B). The intercept represents the systematic variation in PSE when the slope growth factor is set to a time score of 0, which is the baby1 condition for both models. The slopes for model 1 and 2 reflect the systematic part of increase in PSE, from baseline to baby1, and from baby 1 to baby 2 respectively (for interpretations of intercept and slope growth factors, see Muthén & Muthén [40]). In the growth models, it was tested whether the intercept and slope variances, represented as var(i) and var(s) in Fig 1, were significantly different from 0. In order for the model to be identified for two time points, constraints were necessary. In both model 1 and 2, the residual variances (var(e1) and var(e2) in Fig 1) for the PSE measurement occasions were constrained to be equal. In addition, covariances between intercept and slope were constrained to be zero (and are therefore not included in Fig 1). This led to a just-identified model, in which three parameters were estimated: an intercept variance, a slope variance and one residual variance. To check whether demographic variables (age, educational level, single status and ethnicity) had to be included as time-invariant covariates, the intercept and slope of PSE were regressed on the covariates in separate models. The final models only included demographic covariates that were both associated to the independent variable (abuse) and to (one of the) dependent variables (intercept and/or slope of PSE).

Second, for those intercept and slope variances that were found to be significant in step 1, the time-invariant covariate “abuse” (reported abuse experiences with parents in childhood) was added to the model to test whether abuse explained part of the intercept and slope variances. The predictor abuse was coded as a dummy variable with 0 representing no abuse and 1 representing abuse. Next, the abuse variable was divided in three groups (no abuse, abuse unresolved and abuse not unresolved). To examine the association of the unresolved abuse category with PSE, two dummy variables were created. The first dummy represented no abuse (code of 0) vs abuse unresolved (code of 1) and the second represented no abuse (code of 0) vs abuse not unresolved (code of 1). To compare the model fit of the two nested models with and without differentiation of the abuse variable into the unresolved and not unresolved category, the difference in deviance was calculated, which is chi-square distributed with degrees of freedom equal to the difference in parameters between the models.

## Results

### Preliminary analyses

Sixty participants (25%) reported childhood abuse with (one of) their parents or caregivers, of whom 45 participants reported physical abuse, 5 participants sexual abuse, 3 participants both physical and sexual abuse, and 7 participants other forms of abuse. Fifteen participants were classified as unresolved with respect to childhood abuse. With respect to demographic characteristics, women who reported abuse by (one of) their caregivers in childhood were significantly younger (*M* = 26.22, *SD* = 6.51) than women who reported no abuse (*M* = 28.82, *SD* = 5.42; *t* (87.37) = 2.79, *p* = .006). Women who reported childhood abuse also had a lower educational level (*M* = 3.28, *SD* = 1.12) than women who reported no childhood abuse (*M* = 3.87, *SD* = 1.07; *t* (241) = 3.65, *p* < .001). In addition, women who reported childhood abuse were more often single, χ^2^ (1) = 8.85, *p* = .003, and more often had a non-Western background, χ^2^ (2) = 14.50, *p* < .001, compared to women who did not report childhood abuse.

Table 1 presents descriptive statistics of PSE measurements during the Cry Response Task, for the entire sample and separately for the abuse and the no abuse group. Independent sample *t*-tests revealed that women who reported abuse experiences in childhood did not differ with respect to their mean PSE level at baseline or after the easy-to-soothe infant (baby1) from women who reported no abuse experiences, *t* (241) = 0.63, *p* = .526 and *t* (82.36) = 0.19, *p* = .824 respectively. However, women from the abuse group scored lower on PSE after the difficult-to-soothe infant (baby2), *t* (241) = 2.14, *p* = .033. With respect to differences in variances between the abuse and the no abuse group, Levene’s test revealed a significant difference for PSE baby1 only, *F* = 5.09, *p* = .025, indicating that women who reported abuse had more individual variability in PSE after listening to the easy-to-soothe infant compared to women with no reported abuse experiences. This effect was influenced by a few participants in the abuse group with low PSE baby1 values (see ranges for PSE baby1 separately for the abuse and the no abuse group in Table 1). For the analyses of the current study, non-winsorized scores were used for PSE at baseline, PSE baby1 and PSE baby2. However, results were similar if outliers were winsorized, that is replaced by a raw score equivalent to a *z*-score of ±3.29.

**Table 1:** Descriptive Statistics for PSE Measurements during the Cry Response Task.

	Entire sample				No abuse experiences				Abuse experiences			
	*n*	*M*	*Var*	Range	*n*	*M*	*Var*	Range	*n*	*M*	*Var*	Range
PSE baseline	243	69.17	236.16	14–100	183	69.54	242.23	25–100	60	68.08	219.84	14–94
PSE baby 1	243	71.67	188.02	19–100	183	71.79	159.76	38–100	60	71.33	278.23	19–98
PSE baby 2	243	59.04	303.56	5–100	183	60.40	279.49	7–100	60	54.90	359.75	5–93

PSE = parenting self-efficacy

### Model 1: Changes in PSE from baseline to baby1

The intercept mean for PSE baby1 was 71.68, *SD* = .88, *p* < .001. With respect to growth, PSE increased on average (mean slope) from baseline to baby1, *M* = -2.50, *SE* = .74, *p* = .001. In addition, there was significant variation around the intercept of PSE and significant variation around the slope of PSE, ơ^*2*^ = 144.50, *SE* = 16.35, *p* < .001 and ơ^*2*^ = 47.94, SE = 20.00, *p* = .017 respectively, providing support for individual differences around the average intercept level of PSE and individual differences with respect to changes in PSE from baseline to the easy-to-soothe infant. Separate models in which intercept and slope were regressed on each demographic variable revealed that only age was significantly associated with the slope of PSE (not to intercept PSE). PSE increased less in women who were older from baseline to baby1, *b* = .29, *SE* = .13, *p* = .020. Because age was also associated with abuse (see preliminary analyses), age was added to the final model as a covariate.

**Fig 2:**
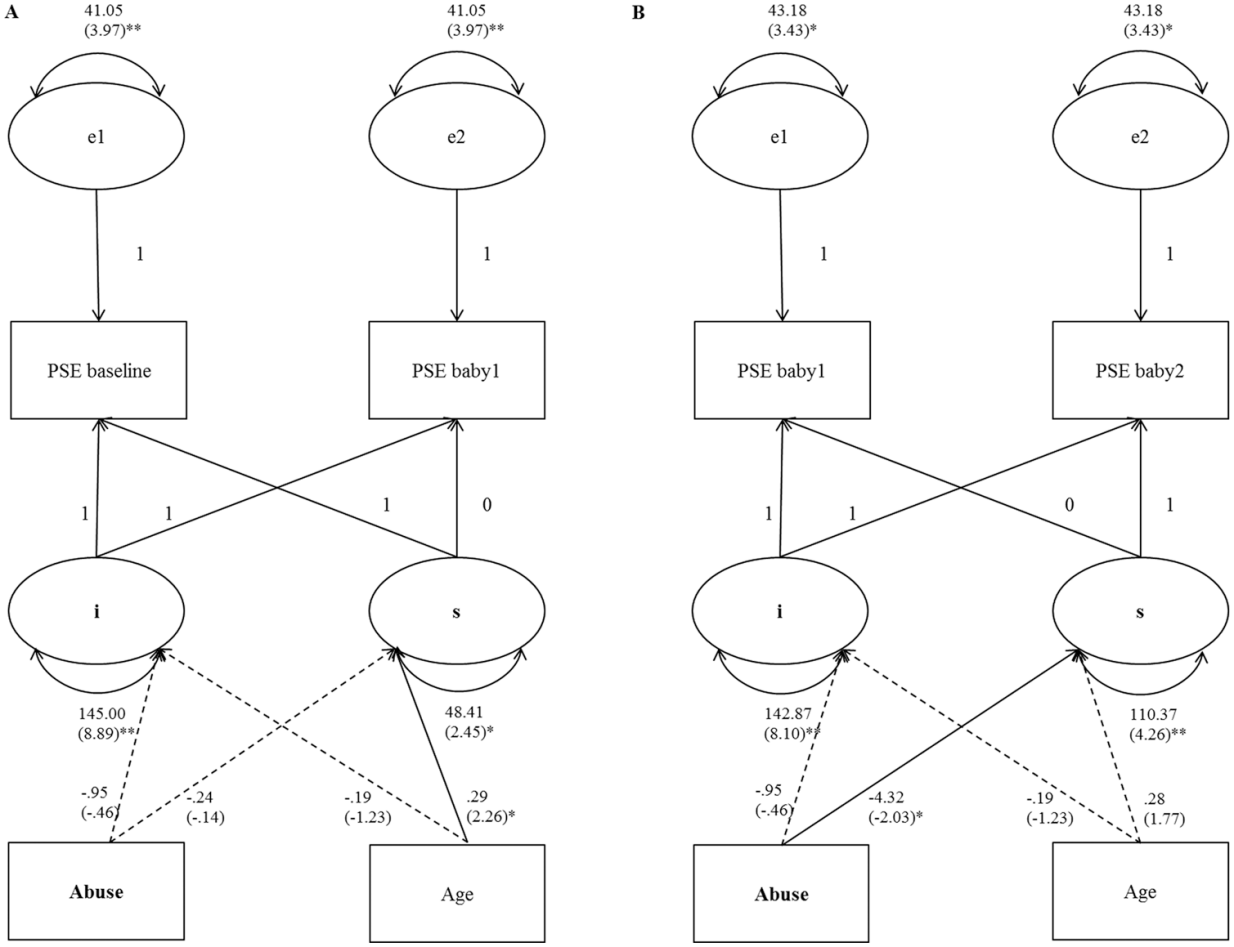
Results of fitted latent growth curve models for PSE during the Cry Response Task with abuse as predictor. (A) Model 1: Predicting changes in PSE from baseline to baby 1. (B) Predicting changes in PSE from baby1 to baby2. PSE = Parenting Self-Efficacy; e1 and e2 = residuals; i = intercept; s = slope. Standardized path coefficients (*z*-scores) are provided in parentheses. * *p* < .05. ** *p* < .001.

Next, effects of abuse on intercept and slope were added to examine whether reported abuse experiences could explain the significant variation in the intercept and slope of PSE. See Fig 2A for the results. Abuse was not significantly related to intercept, *b* = -.95, *SE* = 2.07, *p* = .648, or slope of PSE, *b* = -.24, *SE* = 1.73, *p* = .889. This showed that women who reported abuse did not differ on both PSE level and changes in PSE from baseline to the easy-to-soothe infant from women who did not report abuse. Differentiation of the abuse variable into the unresolved and not unresolved category, did not improve the model fit over the model that included abuse, χ^2^ (2) = 1.55, *p* = .461.

### Model 2: Changes in PSE from baby1 to baby2

A significant mean slope revealed that women decreased significantly in PSE after exposure to the difficult-to-soothe infant, *M* = -12.63, *SE* = .92, *p* < .001. The variances for both intercept and slope were also significant, ơ^*2*^ = 142.79, *SE* = 17.80, *p* < .001 and ơ^*2*^ = 115.07, *SE* = 26.56, *p* < .001 respectively, providing support for individual variability around PSE level and in changes of PSE from the easy-to-soothe infant to the difficult-to-soothe infant. Of the demographic variables again only age significantly predicted the slope of PSE (not the intercept), indicating that PSE decreased less in older women from baby1 to baby2, *b* = .34, *SE* = .16, *p* = .029. Therefore, age was again included as a covariate in the final model.

Given the significance of both intercept and slope variation, both the intercept and slope factors were regressed on abuse. The results are showed in Fig 2B. In this model, age was not significantly predicting the slope of PSE anymore, *b* = .28, *SE* = .16, *p* = .077. The effect of abuse on the intercept of PSE was not significant *b* = -.95, *SE* = 2.07, *p* = .648, but the effect of abuse on the slope of PSE was significant, *b* = -4.32, *SE* = 2.13, *p* = .042. This indicated that women who reported childhood abuse with (one of) their caregivers decreased more in PSE after exposure to a difficult-to-soothe infant than women who reported no childhood abuse. As was observed in scatterplots, one participant who experienced childhood abuse decreased extremely in PSE from baby1 to baby2. By removal of this participant the effect of abuse on the slope of PSE was still significant, *b* = -4.01, *SE* = 2.03, *p* = .049. Again, a comparison of nested models with and without further differentiation of the abuse variable into the unresolved and the not unresolved category yielded no significant difference in model fit, χ^2^ (2) = 1.45, *p* = .485, indicating that a model including this differentiation did not lead to an improved fit to the data. The effects of abuse unresolved (vs no abuse) and abuse not unresolved (vs no abuse) on slope were respectively *b* = -6.02, *SE* = 3.81, *p* = .114, and *b* = -3.52, *SE* = 2.38, *p* = .139.

## Discussion

This study examined whether women who reported abuse in childhood adapted in less resilient ways to challenges to their sense of parenting competence due to infant difficult behavior than women who reported no childhood abuse. In line with our expectations, pregnant women who reported childhood abuse decreased more in PSE in response to the difficult-to-soothe infant (i.e., failure condition) than pregnant women who reported no abuse, whereas no differences were found in women’s adjustment of PSE to the easy-to-soothe infant (i.e., success condition) or with respect to PSE at baseline. These results help to further guide the interpretation of associations found between parents’ own childhood experiences and postpartum PSE in previous studies [7]. Abused women’s postpartum feelings of parenting competence may especially decrease when confronted with challenges and difficulties in parenting their own baby. In particular when exposed to infant difficult temperamental characteristics, such as low soothability, women who experienced childhood abuse may lose faith in themselves as parents more easily than women not reporting childhood abuse.

Results indicated that PSE—on average—increased if women mainly experienced successes, and decreased if women mainly experienced failures in soothing a crying infant (see also [19]), but individual differences in change of PSE across the experiment were observed. Childhood abuse experiences could partly explain individual differences related to changes in PSE in response to failures in soothing, not in response to successes in soothing. This result confirms Bandura’s suggestion [2] that it is potentially more difficult to manage demands or setbacks associated with early parenthood for people who missed effective models in childhood. The failure experiences associated to infant difficult temperament (i.e., low soothability), may particularly confirm pre-existing feelings of low self-worth and incompetence in women who experienced a more negative childhood (see also [20]), which may lead to stronger decreases in PSE. Alternatively, abused women may show stronger emotional and physiological reactivity to infant crying, which may also relate to a further declines in parenting self-efficacy [18].

The finding that reported childhood abuse is related to PSE resilience is in line with attachment theory in that people with adverse early experiences are thought to be more prone to develop negative parental attitudes and less positive expectations about themselves and others [13, 41]. As Bowlby [41] argued mothers who had a difficult childhood may be particularly vulnerable to a baby’s difficult temperament and are therefore more likely to set up a vicious circle of interaction with their temperamentally difficult infant. Our results suggest that abused women’s more negative sense of their own parenting competence (PSE) is one of the potential starting points of this negative spiral resulting in less sensitive caregiving and less positive child outcomes [1].

Individual differences in women’s adjustment of PSE to the difficult-to-soothe infant remained significant after taking into account abuse and age as predictors, which indicates that additional determinants may be important as well in explaining variation in women’s responses. An example of such a determinant may be women’s mood state, which was found to be associated with women’s general sense of parenting competence in previous studies [17]. The individual differences in changes of PSE from baseline to the easy-to-soothe infant may also be explained by ceiling effects, which may have prevented more growth in PSE for women who started off with a high baseline level of expected parenting competence.

Further differentiation between women with unresolved abuse and women with abuse but without unresolved representations did not lead to better prediction of PSE change in response to the difficult-to-soothe infant. Although the effects for the unresolved and not unresolved group both pointed in the same direction (more decrease than the no abuse group), it cannot be excluded that subtle differences may be detected with more statistical power. In order to increase statistical power, future studies should preferably consist of at-risk samples with even larger extreme groups including more people with unresolved abuse experiences. Another possibility is that the non-significant finding fits into the view that reported early adverse experiences and unresolved attachment representations are separate concepts that may yield partly independent effects [42]. In a recent study, reduced hippocampal volume, indicative of vulnerability to mild stress (i.e., hyper- or hyporegulation), was for example only found in adults who reported childhood abuse experiences, not in adults with unresolved attachment classifications [42]. With respect to this study, an unsuccessful response to a salient task such as soothing a crying infant may already be dysregulating for pregnant women who experienced abuse in childhood, regardless of the unresolved/not unresolved status of abuse experiences.

Several potential mechanisms may underlie the association between childhood abuse experiences and a greater vulnerability to difficult infant behaviors. Future research could for example be focused on different ways in which women who report or do not report childhood abuse process a demanding parenting task [9, 43]. Women with a higher potential to abuse their children were for example more physiologically reactive in response to baby cries [22], reported more hostile and negative feelings to the cries [44] or attributed caregiving failure to their own lack of personal control and to a heightened control in their children [45]. Reactions to infant cries, as well as attributions of difficult infant behaviors may be linked to feelings of parenting efficacy [19, 46]. In addition to task-specific features, other mediators or moderators, such as depressive symptoms, self-esteem, partner support, or women’s current relationships with parents, could possibly explain the link between childhood abuse experiences and PSE as well [6, 8, 47]. Specific to the resolved/unresolved classification, this study was a first exploration with regard to the responses of individuals with unresolved and resolved abuse experiences to infant crying. It would be interesting for future studies to include women’s secondary classifications (secure/autonomous; dismissing; preoccupied) in conjunction with unresolved states of mind, because unresolved insecure mothers may be even more vulnerable with respect to their parenting perceptions [48], although such studies would require even larger samples than the current sample, which is already among the largest in the attachment field.

There are several limitations with respect to this study. Although the Cry Response Task with controlled cry stimuli facilitated comparison between (abused and non-abused) pregnant women, it still needs to be examined whether abused women’s responses during this task are also predictive of the way they react to their own infant’s cries. Furthermore, the Adult Attachment Interview with its accompanying coding system was used as a screening instrument for abuse. While an advantage of using this measure was that we could ask for more detail if the occurrence of abuse was unclear, it limited the measured abuse experiences in this study to mainly physical and sexual abuse. Last, statistical power was insufficient to test for specific characteristics of the abuse experiences, such as type of abuse, abuse onset, and who was the abuser.

This study showed that pregnant women who experienced abuse by attachment figures in childhood decreased more in their sense of parenting competence in response to failures to soothe a crying infant. Although at-risk women’s differential responses to infant temperamental difficulty (i.e., low soothability) should still be replicated in more naturalistic studies, these findings do suggest that women who reported childhood abuse may be at increased risk to develop maladaptive parenting cognitions, which may, in turn, negatively affect parental sensitivity and child outcomes as well [1]. In line with results from this study, it is therefore important for health care practitioners to already start in pregnancy with informing at-risk women about the successes as well as failures that go hand-in-hand with early parenthood.
